# Empowering the Physiotherapy Profession in Ethiopia through Leadership Development within the Doctoring Process

**DOI:** 10.3389/fpubh.2017.00051

**Published:** 2017-03-21

**Authors:** Cheryl Burditt Footer, Hailu Seifu Tsegaye, Tesfaye Asnake Yitnagashaw, Wintana Mekonnen, Tizita Destaw Shiferaw, Endashaw Abera, Alice Davis

**Affiliations:** ^1^School of Physical Therapy, Regis University, Denver, CO, USA; ^2^College of Health Sciences, Addis Ababa University, Addis Ababa, Ethiopia; ^3^St. Paul’s Hospital, Millennium Medical College, Addis Ababa, Ethiopia; ^4^Tikur Anbessa Specialized Hospital, Addis Ababa, Ethiopia; ^5^Alert Hospital, Addis Ababa, Ethiopia; ^6^Cure Hospital, Addis Ababa, Ethiopia; ^7^Ethiopian Physiotherapy Association, Addis Ababa, Ethiopia

**Keywords:** Ethiopia, Doctor of Physiotherapy, leadership development, academic partnership, empowerment

## Abstract

Ethiopia recently introduced the Doctor of Physiotherapy (DPT) degree at Addis Ababa University as a mechanism to increase the work force capacity of primary care providers in the health sector. The DPT program was supported by an international academic partnership and was designed to empower physiotherapists as leaders to move the profession forward. The curriculum was framed by core pedagogical principles and strategies and was phased into two programs. First, the 4-year Advanced Standing DPT program focused on developing registered Ethiopian physiotherapists with Bachelor of Science degrees as academic faculty. Second, these new faculty would then sustain a 6-year Generic DPT program that would matriculate students upon graduation from high school. The curriculum represented depth and breadth of foundation and clinical sciences, evidence-based practice, clinical reasoning skills, and interprofessional education opportunities. A leadership thread provided opportunities to develop skills necessary to effectively navigate and manage the challenges faced by the profession. The main outcomes included (1) an 8-year international partnership, (2) the academic performance of students, and (3) and leadership capabilities as demonstrated through activities and assignments. While the program has been criticized as an unnecessary extravagance for Ethiopia, the advantages of the DPT degree were revealed in a direct comparison to other academic physiotherapy programs in Ethiopia. In the end, because the DPT is new to the country, it will take time to fully understand the true impact within the Ethiopian health system.

## Physiotherapy (PT) Profession: Historical Perspective

The PT profession in Ethiopia is relatively new when compared to the history of the profession in other countries (1). The modern era of the PT profession is thought to have begun in Europe toward the end of the nineteenth century. The early origins of physical therapy practice began in hospital-based settings to address the rehabilitation needs resulting from World War I causalities and for individuals impacted by disease epidemics, like poliomyelitis. Today, PTs manage patients with a broad array of health conditions and practice in many different settings, including hospitals, rural health outposts, community-based rehabilitation homes and centers, private and public outpatient clinics, public K-12 schools, pediatric and geriatric settings, and specialized rehabilitation centers (2). The progression of responsibilities and educational standards for entry into the profession has paralleled this expansion. The profession has moved from teaching technical skills, like exercise and massage, to evidence-based strategies for examination, evaluation, diagnosis, prognosis, and intervention of individuals with multisystem health conditions. More than 100 years later, the profession has transformed its entry-level education from short technical-training programs with highly circumscribed knowledge and skill sets to more comprehensive and rigorous professional education programs that culminate in undergraduate and graduate degrees. While not all countries regulate curriculum, some national associations and the World Confederation for Physical Therapy (WCPT) have established guidelines for entry-level education requirements (3). Throughout the world, entry-level degrees span 2-year post-high school diplomas, to 3- and 4-year Bachelor of Science (BSc), 2-year Master of Science (MSc), and 3- and 4-year Doctor of Physiotherapy (DPT). Historically, African nations have offered primarily diplomas and have struggled to upgrade the diploma to a degree program (4). Ethiopia is an exception with the existence of BSc, MSc, and DPT degree options.

## BSc and MSc Degree Programs: Gondar University

At the beginning of the twenty-first century, Ethiopia had only13 PTs registered to serve an estimated 15 million Ethiopians living with disabilities (5). In response to the critical shortage of PT workforce capacity, volunteers from the Netherlands and a team of Ethiopian physicians pioneered the Department of Physiotherapy at University of Gondar. The program sought “to be the best Physiotherapy Department in terms of education, health care and research in East Africa by the year 2020.” They defined their mission as, “committed to the sustainable socioeconomic development of the country achieving our goals through the provision of societal needs tailored education, conducting problem based research and delivering preventive, and rehabilitative services in the field of physiotherapy.” The 4-year BSc program continues to be supported by international faculty from India and the Netherlands. The first cohort of PTs graduated in 2006, with a total estimated graduation of 223 PTs as of 2016 (6).

The BSc curriculum (Table 1) integrates most of the basic foundational technical skill sets for entry-level education set forth by the WCPT (3). However, there is no apparent structure to support evidence-based practice, leadership development, critical thinking, problem solving, and professional judgment skill sets that are recognized as current best practices within the profession worldwide. As a mechanism to further advance the profession, Gondar University initiated a 2-year MSc in Physiotherapy degree in 2009. The MSc curriculum expanded the knowledge base in the content areas of neurologic, orthopedic, pediatric PT, and research methods (Table 1). The MSc program generally accepts a maximum of four BSc prepared PTs each year. To date, the MSc program has graduated an estimated 25 PTs and is supported by international faculty from the University of South Australia and the Netherlands (6).

**Table 1:** **Gondar University 4-year BSc (152 CH/258 ECTS) and 2-year MSc curriculum (38 CH/65 ECTS)**.

BSc	Semester I	Semester II
Year 1	General anatomy	Neuromusculoskeletal anatomy
	Physiology	Orthopedic and general surgery
	Communication skill	Introduction to biomechanics
	Biochemistry	Applied anatomy
	Civic education	Pathology
	Introduction to physiotherapy	Therapeutic exercise I
	Introduction to general psychology	Exercise physiology
	Introduction to sociology	Introduction to rehabilitation service

Year 2	Physiotherapy assessment and treatment I	Physiotherapy assessment and treatment II
	Clinical neurology	Clinical cardiopulmonary condition
	Therapeutic exercise II	Pediatric rehabilitation
	Electrotherapy	Neurological rehabilitation
	Physiotherapy clinical practice I	Massage therapy
	Clinical pediatric conditions	First aid
	Introduction to general psychology	Introduction to general psychology

Year 3	Cardiopulmonary pulmonary rehabilitation	Introduction to biostatics
	Physiotherapy in OBG conditions	Introduction to epidemiology
	Pain management	Occupational and environmental science
	Prosthetics, orthotic and mobility aids	Introduction to health informatics
	Community-based rehabilitation	Nutrition
	Introduction to pharmacology	Radiology
	Health ethics	Physiotherapy clinical practice III
	Sports physiotherapy	
	Physiotherapy clinical practice II	

Year 4	Physiotherapy in geriatric	Physiotherapy clinical practice V/internship practice
	Teaching skill (methodology)	Physiotherapy clinical practice VI/internship practice
	Health service management	
	Research methodology	
	Research project	
	Physiotherapy clinical practice IV	

**MSc**	**Semester I**	**Semester II**

Year 1	Library and information technology	Musculoskeletal physiotherapy
	Basic biostatistics, evidence-based practice, and critical review	Musculoskeletal physiotherapy clinical practice
	Teaching skills	Physiotherapy trauma and orthopedics
	Leadership, management, project planning	Trauma and orthopedics and clinical practice
	Ethics, clinical governance, and audit	
	Biostatistics II and research methods	

Year 2	Neurological physiotherapy	Research dissertation
	Neurological physiotherapy clinical practice	
	Pediatric physiotherapy	
	Pediatric physiotherapy clinical practice	

*BSc, Bachelor of Science; MSc, Master of Science; ECTS, European Credit Transfer and Accumulation System*.

## Challenges for PTs in Ethiopia

As with the introduction of any new health profession, Ethiopian PTs have met professional challenges within the health sector, which has impacted the general motivation by some to pursue or continue a career in PT. Thus, despite the development of the University of Gondar programs, Ethiopia continues to experience limitations in human resource capacity to meet the demand for rehabilitation services. There are a number of potential causes for the limitations.

First, there is a lack of overt public and government support for the physical therapists, possibly due to a limited understanding of this relatively new Ethiopian profession. Second, the Ministry of Education assigns post-high school students to the academic program based on their interests in health care and on their performance on the Ethiopian Higher Education Entrance Certificate Examination. It is perceived that some of those assigned to PT programs would prefer to seek opportunities in other health professions. Third, while Ethiopian physicians have opportunities for continued professional development, the PT profession has extremely limited opportunity in this area. Thus, the growth and development of individual practitioners and the profession as a whole is limited by this void. Fourth, poor salary compensation may cause PTs to seek additional ways to supplement their income, or they choose to leave the profession altogether. Fifth, a shortage of qualified local PT faculty limits the ability to sustain the academic programs without international support. In addition, local faculty may not be fully qualified for academic roles as they often receive little to no formal training in teaching pedagogy, nor advanced PT skill sets to optimally integrate into the academy. Finally, Ethiopia has a broad Scope of Practice for the PT profession that is dependent upon the level of academic preparation. This leads to disparities in the delivery of patient care among PT providers.

The sum of the limitations greatly impacts the profession throughout the country. Therefore, the focus of this special topic paper is to describe the development of the DPT program at Addis Ababa University (AAU) as a potential strategy to alleviate the impact of the many limitations on the PT work force capacity in Ethiopia.

## DPT Degree Programs: AAU

**Figure 1:**
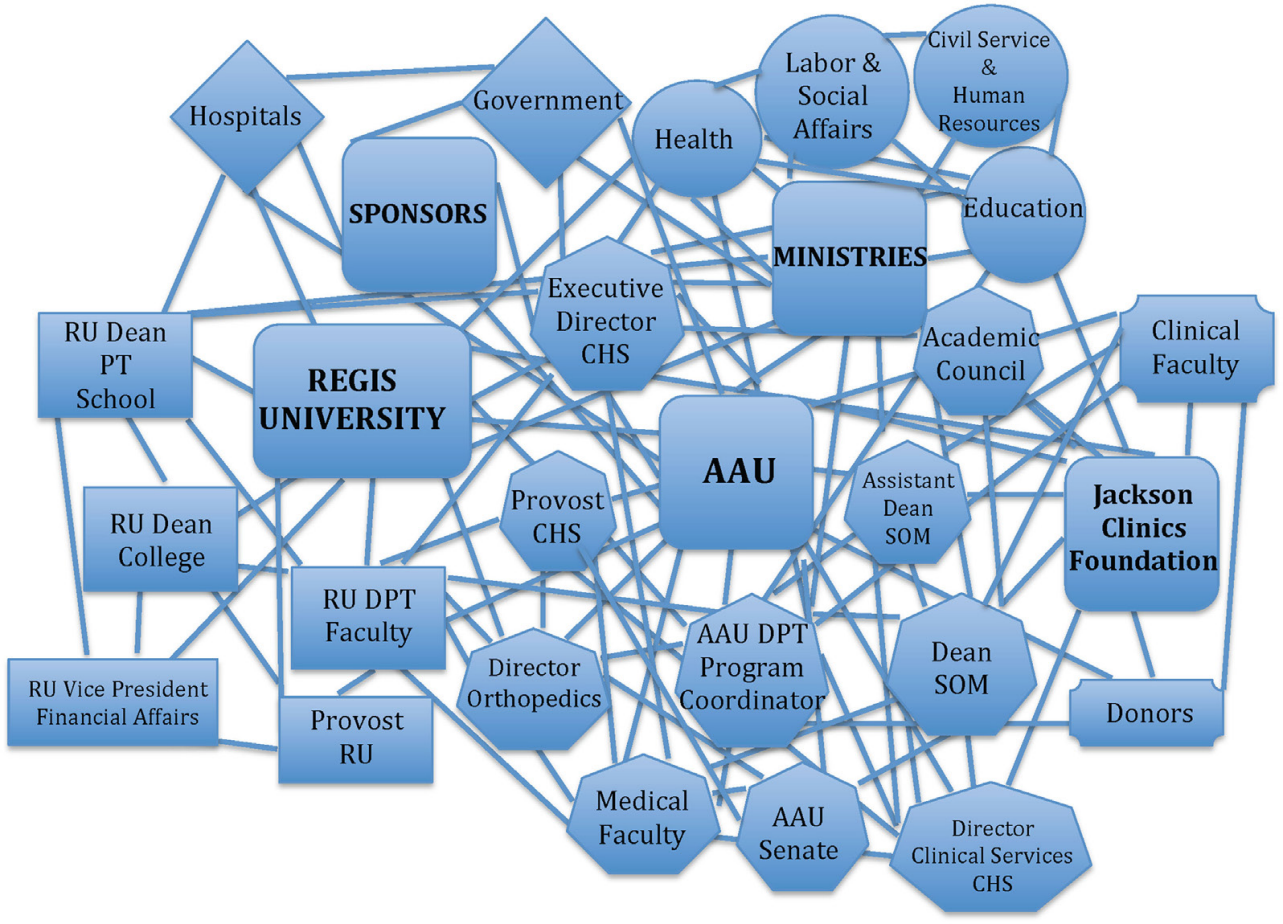
**Web of partner relationships for the development of the DPT program at AAU**. DPT, Doctor of Physiotherapy; AAU, Addis Ababa University; CHS, AAU College of Health Sciences; SOM, AAU School of Medicine; RU, Regis University.

The DPT was introduced to Ethiopia at AAU in 2008. The program defined the vision to “be recognized in Sub-Saharan Africa as a center of excellence for education and service provision by doctors of physiotherapy. These doctors will be physical medicine experts who deliver optimal patient care and contribute to the goals of the health sector.” The development of the DPT program was planned in a partnership between AAU, Regis University (Denver, CO, USA), the Jackson Clinics Foundation, LLC (Middleburg, VA, USA). Over the 6-year planning period, the partnership developed relationships with several Ethiopian stakeholders that played critical roles in the formation and approval of the DPT program at AAU (Figure 1). The partners encountered many challenges during this planning phase, mostly related to the frequent replacement of the AAU College of Health Science and School of Medicine Deans and Directors and the accompanying breakdowns in communication between outgoing and incoming administrators. Despite the challenges, the AAU Senate ultimately approved the Advanced DPT Standing program in 2014 and matriculated the inaugural cohort of 17 registered PTs shortly thereafter.

The introduction of the DPT at AAU was designed to occur in two phases as follows: (1) the Advanced Standing DPT Program and (2) the Generic DPT Program.

### Phase 1: Advanced Standing DPT Program

The Advanced Standing DPT program was developed with consideration of the (1) social and economic development level of the country, (2) analysis of history, development, and present status of the PT profession in Ethiopia and in other countries, (3) educational sector development plan of Federal Democratic Republic of Ethiopia (FDRE), (4) health sector development plan of FDRE, (5) standards of admission, program design, licensing, and accreditation needs for the education of PT professionals, and (6) new developments in research within the profession of PT. The scope of the 4-year Advanced Standing curriculum (Table 2) was substantially greater than the Gondar BSc curriculum and was tailored to match the experience of the registered PTs and to specifically prepare them for professional roles as faculty of the Generic DPT program (see below). The graduation of the first cohort of Advanced Standing students is anticipated in May 2018. They will officially assume their roles as core and/or clinical faculty for the Generic DPT program in Fall 2018.

**Table 2:** **Addis Ababa University 4-year Advanced Standing DPT curriculum (147 CH/249 ECTS)**.

	Semester I	Semester II
Year 1	Neuromusculoskeletal anatomy	Exercise prescription
	Human development	Cardiovascular and pulmonary system management
	Exercise physiology	Pharmacology: cardiovascular and pulmonary
	Kinesiology	Evidence-based practice: cardiovascular and pulmonary
	Clinical decision making	Clinical mentoring I: cardiovascular and pulmonary
		Movement science
		Movement analysis

Year 2	Qualitative and diagnostic EMG	Radiology
	Neurological management I	Musculoskeletal management I: LE
	Neurological management II	Musculoskeletal management II: UE
	Pharmacology: neurology	Evidence-based practice: orthopedics-extremities
	Evidence-based practice: neurology	Orthopedic trauma medicine
	Clinical mentoring II: neurology	Clinical mentoring III: orthopedics-extremities

Year 3	Musculoskeletal management III spine	Roles and responsibilities
	Pharmacology: orthopedics	Practice management
	Evidence-based practice: orthopedics-spine	Health policy
	Therapeutic exercise	Community and rural health services
	Clinical mentoring IV: orthopedics: spine	Emergency medicine
	Physical medicine	Clinical mentoring V: population health
	Sports medicine	

Year 4	Endocrinology/integumentary	Pediatrics
	Evidence-based practice: endocrin/integ	Adult
	Clinical mentoring VI: endocrin/integ	Geriatrics
	OB/GYN	Clinical mentoring IX
	Evidence-based practice: OB/GYN	
	Clinical mentoring VII: OB/GYN	
	Oncology	
	Evidence-based practice: oncology	
	Clinical mentoring VIII: oncology	
	Research methods	

*DPT, Doctor of Physiotherapy; ECTS, European Credit Transfer and Accumulation System*.

### Phase 2: Generic DPT Program

The Generic DPT program was designed as a 6-year interprofessional (IPE) program within the AAU School of Medicine. The curriculum was expanded from the Advanced Standing program by incorporating the first 2 years of the medical school foundational curriculum (Table 3).

**Table 3:** **Addis Ababa University (AAU) 6-year Generic Doctor of Physiotherapy (DPT) curriculum (231 CH/393 ECTS)**.

	Semester I	Semester II
Pre-year 1	Medical psychology^a^	
	Information technology/computer application^a^	
	Medical sociology^a^	
	Civics and ethical education^a^	
	Communicative English skills^a^	
	Basic English writing skills^a^	

Year 1	Body structure, organization and functions^a^	Blood and immunity^a^
	Metabolic homeostasis/molecular genetics^a^	Neoplasia and molecular basis of cancer^a^
	Basic concepts of disease and therapy^a^	Musculoskeletal, integumentary and craniofacial regions^a^
		Epidemiology and biostatistics^a^

Year 2	Cardiopulmonary^a^	Endocrinology and reproduction^a^
	Excretion and toxicology^a^	Neuroscience and behavior^a^
	Nutrition metabolic diseases and GIT^a^	Infectious diseases^a^
	Environmental health^a^	Research methodology^a^

Year 3	Exercise physiology	Exercise prescription
	Kinesiology and biomechanics	Cardiovascular and pulmonary system management
	Physiotherapy skills^b^	Pharmacology: cardiovascular and pulmonary
	Physiotherapy examination^b^	Evidence-based practice: cardiovascular and pulmonary
	Clinical decision making	Clinical mentoring I: cardiovascular and pulmonary
		Movement science
		Movement analysis

Year 4	Qualitative and diagnostic EMG	Radiology
	Neurological management I	Musculoskeletal management: I
	Neurological management II	Musculoskeletal management: II
	Pharmacology: neurology	Evidence-based practice: orthopedics-extremities
	Evidence-based practice: neurology	Orthopedic trauma medicine
	Clinical mentoring II: neurology	Clinical mentoring III: orthopedics-extremities

Year 5	Musculoskeletal management III	Roles and responsibilities
	Pharmacology: orthopedics	Practice management
	Evidence-based practice: orthopedics-spine	Health policy
	Therapeutic exercise	Community and rural health services
	Clinical mentoring IV: orthopedics: spine	Emergency medicine
	Physical medicine	Clinical mentoring V: population health
	Sports medicine	

Year 6	Endocrinology/integumentary	Pediatrics
	Evidence-based practice: endocrin/integ	Geriatrics
	OB/GYN	Clinical mentoring VI: summative internship
	Evidence-based practice: OB/GYN	
	Oncology	
	Evidence-based practice: oncology	
	Research methods	
	Prosthetics and orthotics^b^	

*^a^Courses taught by AAU medical school faculty using an interprofessional model with medical students*.

*^b^DPT only courses that were not included in the Advanced Standing program*.

In addition to the benefits cited for the Advanced Standing DPT program, the Generic DPT program will promote IPE collaboration in the classroom and in clinical practice and, thereby, expand potential for improved patient outcomes. Ultimately, the Generic DPT program will be fully sustained by PT faculty developed in the Advanced Standing program in coordination with the AAU medical school faculty. International faculty will not be needed to sustain the Generic DPT program. AAU plans to matriculate the first cohort of Generic DPT students in Fall 2017.

## Pedagogical Principles and Framework for the DPT Curriculum

### Core Pedagogy Principles

The development of the DPT curriculum was guided by six core pedagogical principles that emphasized empowerment, leadership, relationships, active engagement, reflection, and innovation.

Principle 1: empowerment through learning is the path to the future.
Principle 2: leaders dwell within everyone.
Principle 3: relationships form the basis of all learning.
Principle 4: students achieve growth by challenging the process and modeling the way.
Principle 5: active engagement amplifies learning achievement.
Principle 6: reflective processes inspire innovation.

### Pedagogy Framework

#### Doctor of Physiotherapy

**Figure 2:**
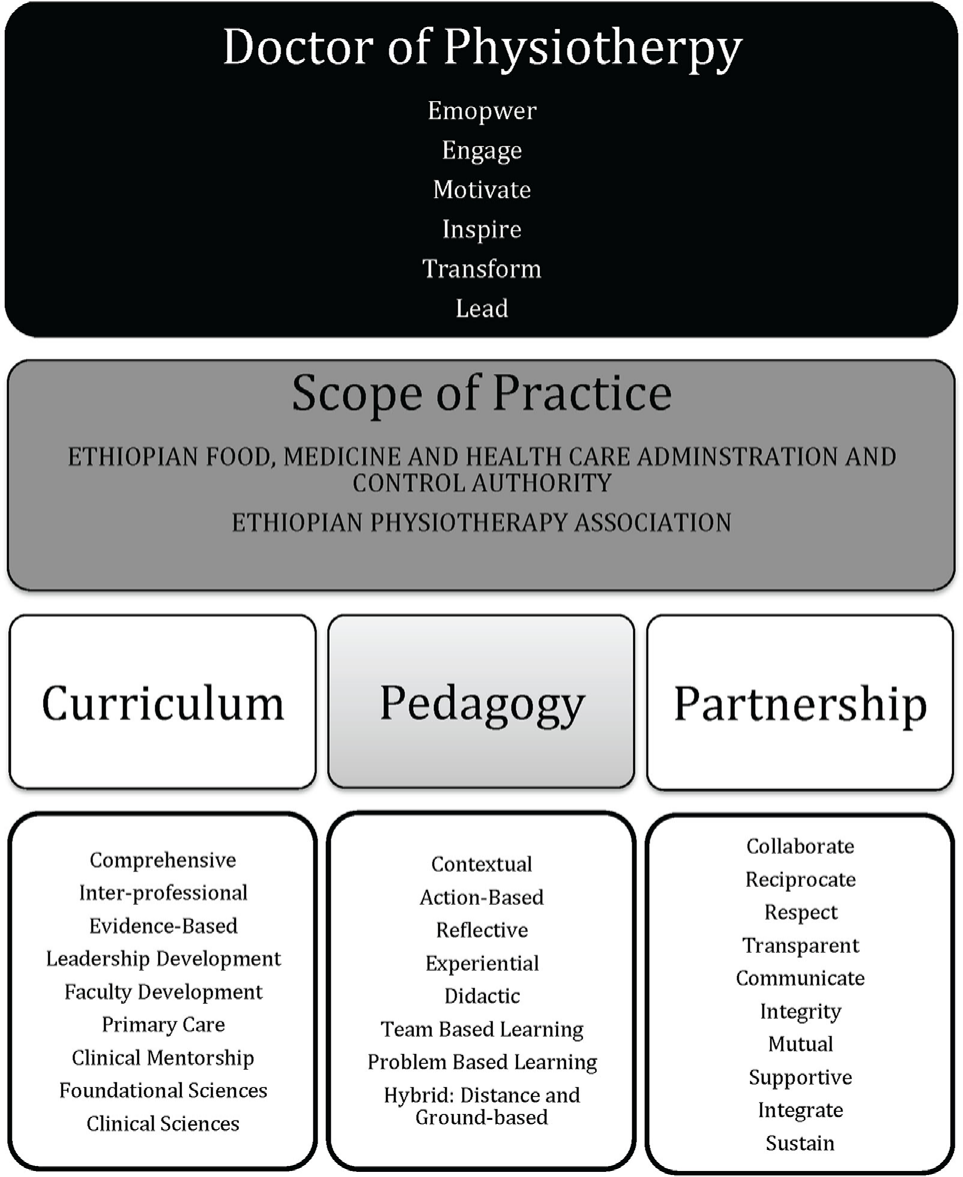
**Pedagogical framework for the Doctor of Physiotherapy programs**.

The pedagogical framework was designed to build upon the six core principles while acknowledging the local context of the physical medicine and rehabilitation needs of Ethiopia, the Scope of Practice, and the constraints of a resource-limited health environment (Figure 2). The DPT student was the primary focus of the framework, which aimed to empower, engage, motivate, inspire, transform, and develop leaders for the profession.

#### Scope of Practice

The framework required a thorough understanding of the vision of the PT profession in Ethiopia and was, thus, guided by the Scope of Practice. The Ethiopian Physiotherapy Association and the Ethiopian Food, Medicine and Health Care Administration and Control Authority jointly wrote the Scope of Practice. The academic degree determines the scope of duties that a PT can perform, e.g., technician, BSC, MSc, and DPT. Ethiopian DPT practitioners have the highest capacity of all academic PT degrees to deliver primary care services.

#### Pedagogical Format and Curriculum

As previously stated, the Advanced Standing DPT and Generic DPT programs curricula were specifically designed to meet the unique needs of Ethiopia. Ethiopia aimed to increase the workforce capacity by including PTs as primary care providers in the health system. Thus, the DPT curriculum had to expand its scope beyond the BSc and MSc to meet the expectations and responsibilities that accompany the provision of primary care. For example, Ethiopian DPTs needed training to order medically relevant diagnostic tests, participate in the management of medical/surgical emergencies or trauma, perform specialty interventions such as mechanical decompression, plastering techniques, joint and soft tissue injections, prescribe neuromusculoskeletal medications, perform minor trauma suturing, reduce and stabilize minor fractures and dislocations, and perform electromyography, lower extremity Doppler tests, and stress tests. The curriculum also included a leadership thread to cultivate and empower leaders for the PT profession. A teaching and learning thread was also woven throughout the curriculum to develop Ethiopian faculty that could independently sustain the academic programs. Finally, the curriculum was taught with IPE collaboration among the faculty and students from other health professions to facilitate networking and teamwork among health professionals.

The curriculum was delivered in accordance with the guidelines for AAU College of Health Sciences, School of Medicine modular undergraduate curriculum. The Advanced Standing DPT program included 51 courses (Table 2) that were sectioned into 18 content modules (see below).

##### Modules

1. Human Anatomy and Development
2. Physiology and Kinesiology of Movement
3. Clinical Decision Making
4. Cardiovascular and Pulmonary System Management
5. Movement Sciences
6. Diagnosis—Electromyography Studies
7. Neurological Systems Management
8. Radiology
9. Musculoskeletal Management I—Extremities
10. Musculoskeletal Management II—Spine
11. Specialized Medical Management of the Musculoskeletal System
12. Professional Roles
13. Population Health
14. Integumentary and Endocrinology
15. Urogenital/OB-GYN
16. Oncology
17. Research Methods
18. Scope of Physiotherapy Practice

All courses were delivered in a blocked design, where students were required to take one course to completion before beginning the next course and subsequent module. The blocked design also accommodated the international faculty travel schedules.

The Generic program will also be taught with a modular system but will be taught with a traditional distributed delivery model rather than a blocked model. In addition, the Generic program will expand the curriculum to 6-years; thus 21 new courses (Table 3) will be sectioned into 12 new modules (see below).

##### Modules

1. Body Structure, Organization, and Function
2. Metabolic Homeostasis and Molecular Genetics
3. Basic Concepts of Disease and Therapy
4. Blood and Immunity
5. Neoplasia and the Molecular Basis of Cancer
6. Musculoskeletal and Integumentary Systems
7. Cardiopulmonary System
8. Nutrition, Metabolic Diseases and GIT
9. Excretion and Toxicology
10. Infectious Disease
11. Endocrinology and Reproduction
12. Neuroscience and Behavior

The additional 12 modules will be taught by AAU medical school faculty and represent the current foundational courses for medical students. The DPT students will take these courses alongside medical students during their first 2 years of the curriculum.

#### Pedagogical Strategies and Learning Environment

The Advanced Standing DPT program employed several pedagogical strategies throughout the curriculum to meet the needs of diverse learners and to expose the students to a variety of strategies that would be available to them as they prepared for their roles as future faculty.

##### Learning Environment

The curriculum was delivered with a hybrid model that utilized ground-based and distance-based delivery models to accommodate the availability of experienced international faculty. Ground-based instruction incorporated both didactic and practical (action-based) skill development and was delivered in a traditional classroom on the AAU School of Medical campus. Distance-based was primarily utilized for the delivery of didactic content and was delivered through a Skype connection into the AAU classroom.

##### Lecture

Traditional lecture formats were utilized throughout the curriculum, but with intentional efforts to actively engage the student in dialog. Non-traditional lecture formats that utilized technology with audio-enhance lectures (flipped classroom concept) were also implemented when a faculty member could not be on-site or when students needed additional course review beyond the physical structure of the classroom.

##### Labs

Many of the courses incorporated lab experiences to teach, learn, demonstrate, and practice practical skills.

##### Small Group Instruction

Clinical mentoring courses for experiential patient management were performed in small groups of 3–4 students. The curriculum included a series of nine clinical mentoring courses. A similar model will be incorporated into the Generic program.

##### Student Demonstration/Instruction

Adopting the model, “See One. Do One. Teach One,” the students were frequently encouraged to assume the role of instructor to gain experience for their future roles as faculty.

##### Team-Based Learning (TBL)

Students completed individual readiness assurance tests and team readiness assurance tests on content assigned for specific days within selected courses. The TBL model encouraged learners to arrive to class fully prepared to engage in teamwork with other students.

##### Problem-Based Learning

Contextual-based case scenarios were adapted for various courses to promote thoughtful reflection, dialog, critical thinking, problem solving, and group process.

##### Assessment

The students were assessed with a variety of methods, including written examinations, quizzes, practical examinations, written assignments, and oral presentations.

#### Partnership

As stated earlier, academic instruction for the Advanced Standing program was supported by an international partnership. The partnership was manifested through a complex network of relationships (Figure 1) and a memorandum of understanding (MOU) that delineated the roles of each partner in supporting the program. In short, all partners had human resource commitments related to administrative roles and teaching responsibilities within the curriculum, as well as fiscal and physical resource commitments. The international partners also committed to providing mentorship to the newly formed faculty upon their graduation but do not plan to formally teach within the academic programs thereafter.

## Competencies and Objectives

The DPT curriculum represented depth and breadth of both foundational and clinical sciences and was accompanied by competencies and objectives reflective of an entry-level DPT curriculum. In addition, a leadership thread was woven throughout the curriculum to develop leadership for the advancement of the profession in Ethiopia.

The leadership goals and objectives were unique to AAU’s DPT program and were not represented within the BSc program at the University of Gondar. They included the following:

1. Illustrate the social responsibilities of a doctoring profession.
2. Advocate for patient and community needs.
3. Commit to meet professional obligations.
4. Consider the determinants that impact societal health and wellness.
5. Adhere to high ethical principles and professional values and standards.
6. Accept responsibility for roles, obligations, and actions of the physiotherapist.
7. Consider social, emotional, and psychological components in patient care.
8. Acknowledge that effective health care depends on a mutual understanding and relationship between the health-care team, patient, and the family with respect for the patient’s welfare, culture, identity, beliefs, and autonomy.
9. Demonstrate patient-centered actions ahead of the self-interest of the physiotherapist by treating patients with politeness and consideration, respecting their dignity, privacy, and point of view without regard to background, culture, language, religion, race, and point of view.
10. Participate in lifelong learning to ensure ongoing high levels of knowledge and clinical competence.
11. Serve as leaders to elevate the standards of PT care in Ethiopia to best meet the needs of society.

## Current Results and Outcomes

### Partnership

As stated previously, the partnership experienced significant turnover within the AAU academic administration. The Executive Director of the college of Health Sciences changed three times, the Academic Dean of the School of Medicine changed four times, the Associate Dean of the College of Health Sciences changed four times, the Director of Undergraduate Medical Education changed three times, and the Director of the Department of Orthopedics (where the Unit of Physiotherapy resides) changed five times. The instability and longevity of the higher education administration made the progression of the program difficult. Fortunately, the commitment of the DPT program Coordinator at AAU remained constant throughout all of the transitions in higher administration to sustain the partnership. All partners remain committed to the progression of the academic programs.

### Generic DPT Program Development

The Generic DPT curriculum is currently in its final stage of development. The curriculum is scheduled for submission to the AAU Senate for review and approval in early 2017. Once it is approved, AAU will begin the process to matriculate post-high school students into the program in Fall 2017.

### Advanced Standing DPT Students

#### Survey

The 17 DPT students that matriculated into the Advanced Standing DPT program are currently completing the third year of the curriculum. To date, 36 courses have been taught by a total of 29 faculty, including 14 different AAU faculty for 18 courses, 7 different Regis University faculty for 15 courses, and 8 Jackson Clinics Foundation clinicians for 3 courses.

An extensive survey was administered to all students at the end of their first year. The survey addressed three primary areas and utilized a 4-point scale; 1 = novice: shows an understanding of the behavior but does not consistently act in this manner; 2 = competent: performs the behavior upon request, but not necessarily in every day activities; 3 = proficient: regularly acts in this manner and can provide recent examples; and 4 = expert: always behaves in this way and can illustrate with many recent examples.

1. Professional behaviors, roles, and responsibilities of the DPT profession: the domains with an average score in the proficiency range (mean = 3.07–3.14; SD = 0.57–61) were leadership, collaboration and relations building, communication, and altruism. The domains in the competency range (mean = 2.55–2.81; SD = 0.57–0.69) were professional roles and behaviors, advocacy, social responsibility, accountability/ethical/legal issues, self-assessment/peer-assessment, education, evidence-based practice, and critical thinking/clinical reasoning.
2. Principles for patient management: the domain with an average score in the proficiency range (mean = 3.01; SD = 0.44) was guiding principles for examination. The domains in the competency range (mean = 2.71–2.97; SD = 0.22–0.61) were guiding principles for diagnostic tests, evaluation, diagnosis, prognosis, plan of care, intervention, outcomes assessment, and education intervention.
3. System and patient-specific management: the domain with an average score in the proficiency range (mean = 3.05; SD = 0.56) was musculoskeletal examination. The domains in the competency range (mean = 2.13–2.99; SD = 0.51–0.88) were cardiopulmonary, integumentary (examination only), musculoskeletal, neuromuscular, orthotic/prosthetic/protective devices, and assistive/adaptive devices. The domains with an average score in the novice range (mean = 1.84–1.90; SD = 0.79–0.85) were integumentary procedural interventions and special populations.

The survey will be readministered at the completion of the 4-year Advanced Standing DPT program, and the results will be analyzed as a component of the overall assessment of the program.

### Leadership Development

The critics of the AAU DPT degree argue that a change in academic degree will not resolve the challenges faced by the PT profession in Ethiopia. Indeed, they are probably right. Taken at face value, the expansion of the curriculum with the DPT degree is evident. However, less obvious was how the leadership thread served as a potential mechanism to address the aforementioned limitations.

The leadership thread was an integral part of the Advanced Standing program and will continue with the Generic DPT program. The thread exposed students to a variety of leadership theories, models, and assessment tools. It offered opportunities to engage in reflective processes and gain perspectives on personal leadership styles as well as the leadership traits of others. Students individually and jointly reflected on their personal and professional values and future roles and responsibilities in the Ethiopian health-care environment. They also deliberated on leadership topics in groups to identify core professional values that they believed could lead the profession forward in Ethiopia. The current cohort identified the core PT values as professionalism, timeliness, accountability, knowledge and skills, and transparency and coupled them with their personal values of compassion, integrity, love, kindness, and honesty. Finally, the cohort worked as a team to develop action verbs to accompany the personal and professional values that would enable them to act upon the identified values. They chose to empower, promote, work, demonstrate, strive, pursue, and engage.

The thread required students to develop personal mission statements that could guide their journey as individual leaders in academia and clinical practice. General themes that emerged from the mission statements included statements to lead the PT profession forward through active involvement in policy making, to inspire and empower current and future PTs, to embrace altruism in practice, to advance the quality of patient care, and to be accountable to the profession and those that they served.

The culminating activity within the leadership thread was the development and implementation of personal leadership projects. The projects were a mechanism to motivate and empower them to address the challenges in Ethiopia by engaging as positive change agents. The leadership projects reflected the added value of the leadership thread within the curriculum. The leadership project themes ranged from being able to more fully integrate PTs as leaders within the Ministry of Health and improving the awareness of the profession in the country to improving PT work efficiencies and physical environments in hospitals and clinics to increase job satisfaction and retention of PTs in the work force. The general feedback about the leadership thread from the current cohort was extremely positive, as many of them believed they could be positive change agents and lead the profession forward as a result of the leadership development process.

## Discussion

Ethiopia has made great advances in developing academic programs to address the shortage of PTs in the workforce. Over the past 15 years, the contributions of Gondar University’s BSc and MSc programs have contributed over 200 PTs to the physical medicine and rehabilitation sector. The DPT programs at AAU were designed to address the mounting challenges of the profession by increasing the scope of the curriculum for primary care and developing leaders to move the profession forward.

The DPT programs have been subjected to criticism, especially since the degree is new to Ethiopia and all of Africa. The harsh opinions have included statements such as:

- “Africa does not need doctoral education. They simply need basic training to meet the general growing needs of the population.” (USAID Representative)
- “Ethiopia does not need autonomous PTs, they need to work with the physicians as a team.” (European Physician)
- “The BSc degree works around the world. Does the world really need the American DPT?” (European PT)

To the critics, it can be argued that the challenges will only continue to grow if innovation is not pursued. Examination of the different curricular models in Ethiopia revealed the advantages of DPT degree compared to the BSc and MSc degree. Benefits to the profession included developing leaders; advancing clinical reasoning skills and application of evidence-based practice beyond the technical skill sets associated with the BSc and MSc programs; broadening the depth of foundational and clinical practice knowledge to fully address the professional scope of practice in primary care; promoting a culture of lifelong learning and service to others; envisioning positive change for the future of the profession; instituting IPE in educational curricula and clinical practice for optimization of patient outcomes; developing local faculty to sustain academic programs; increasing the potential for greater income, as the DPT will be compensated on a doctoral scale like physicians; and improving the potential for greater recognition and sustained increased in work force capacity within the profession. While preliminary data have been collected related to professional behaviors, roles, and responsibilities of the DPT profession, principles for patient management, and system and patient-specific management, future analysis of data from graduation surveys will serve as quantitative markers of the impact of the program.

## Limitations and Recommendations

The greatest limitation of the DPT program at AAU has been the inconsistencies of higher administration support due to the frequent turnover of personnel. It is strongly recommended that the primary coordinator/director of the program have a strong presence in the daily academic environment to ensure stability and sustainability of the program. Limitations were also noted in the availability of AAU medical school faculty and international PT faculty. The MOU designated courses to be taught by the AAU medical school faculty, but it was often difficult to secure the actual commitment of the faculty. The challenge was related to difficulties in communication and expectations between higher administration and the faculty. For almost all of the international faculty, they could only commit to teach in 2-week blocks due to ongoing responsibilities within their home institutions. Students reported that they would have preferred the courses to be taught over a 3- to 4-week timeframe to allow time to fully integrate the course materials. In addition, the expense to send international faculty to Ethiopia was quite high. The opportunities for external funding and grants were essentially non-existent for the DPT program, as most grants/funds directed to Africa targeted HIV/AIDS, TB, malaria, and other medical student education programs. Advocacy for the PT education and profession grants in Africa would be strongly encouraged. Finally, the DPT program at AAU was specifically designed to meet the unique needs of Ethiopia and, thus, the curriculum may not be fully generalizable to other countries. However, the degree has many benefits that warrant careful consideration as a potential mechanism to upgrade the diploma to a degree in Africa. With this in mind, the process by which the DPT was developed in Ethiopia is, indeed, generalizable. The process involved a grassroots movement of Ethiopian PTs, a collaborative partnership for expertise (it would be difficult for any one person to have all the necessary expertise to develop a new academic model in any country), support of internal (academic institution) and external governing bodies (ministries, etc.), and students willing to commit to a pioneering academic program. The process requires time to navigate necessary systems to launch the program. It is very important to be patient. Due diligence to complete all requirements to launch a new program is the ultimate key to facilitating the sustainability and success of the DPT degree.

## Conclusion

Since the program is still very new, it will take time to understand the impact of the DPT in Ethiopia. The only marker that exists at this time is the performance of the 17 students currently enrolled in the Advanced Standing program, all of who graduated from the BSc program at Gondar University. One particular student comment stands out to reflect the value of the DPT program:

I never knew what it was to be a PT until I started the DPT program.

So, the question to be asked is. “Can the DPT make a difference in Ethiopia?” If any credence is to be applied to the empowerment potential of the leaders being developed in the Advanced Standing DPT program, then the answer will hopefully emerge as, “Yes, the DPT can make a difference!”

## Author Contributions

CF, the lead instructor for the Doctor of Physiotherapy program at Addis Ababa University, contributed historical knowledge of program development at Addis Ababa University as one of the international partners and also provided specific details of the history of PT profession development, curriculum design, and pedagogy. AD, the lead instructor for the leadership development component of the Doctor of Physiotherapy program at Addis Ababa University, contributed key knowledge of the curriculum design and pedagogy. HT contributed valued perspective as a student in the doctoral program at Addis Ababa University and also contributed knowledge of the challenges within the Ethiopian health sector. TY, WM, and TS contributed valued perspective as students in the doctoral program at Addis Ababa University. EA provided a voice as the President of the Ethiopian Physiotherapy Association and contributed rich knowledge of the historical perspective and future direction of physiotherapy in Ethiopia.

## Conflict of Interest Statement

The authors declare that the research was conducted in the absence of any commercial or financial relationships that could be construed as a potential conflict of interest.
